# Outcomes of gonioscopy-assisted transluminal trabeculotomy in pseudoexfoliative glaucoma: 24-month follow-up

**DOI:** 10.1136/bjophthalmol-2020-315954

**Published:** 2020-07-29

**Authors:** Eamon Sharkawi, Daniel Josef Lindegger, Paul H Artes, Lydia Lehmann-Clarke, Mohamad El Wardani, Marie Misteli, Jérôme Pasquier, Adriano Guarnieri

**Affiliations:** 1 Swiss Eye Centre, Lausanne, Switzerland; 2 Moorfields Eye Hospital, London, London, UK; 3 University of Plymouth, Plymouth, UK; 4 Southwest Eye Institute, Tavistock, UK; 5 Jules Gonin Eye Hospital, Lausanne, Switzerland; 6 Unisanté, Center for Primary Care and Public Health, University of Lausanne, Lausanne, Switzerland

**Keywords:** Glaucoma, intraocular pressure, treatment surgery

## Abstract

**Aim:**

To report on outcomes of gonioscopy-assisted transluminal trabeculotomy (GATT) in eyes with pseudoexfoliative glaucoma (PXG).

**Methods:**

Prospective, interventional, non-comparative case series. A total of 103 eyes from 84 patients with PXG were enrolled to undergo a 360-degree ab interno trabeculotomy with gonioscopic assistance using either a 5.0 polypropylene suture or an illuminated microcatheter with up to 24 months of follow-up. Main outcome measures were intraocular pressure (IOP), number of antiglaucoma medications, success rate (IOP reduction ≥20% from baseline or IOP between 6 and 21 mm Hg, without further glaucoma surgery) and complication rate.

**Results:**

Mean preoperative IOP was 27.1 mm Hg (95% CI 25.5 to 28.7) using 2.9 (SD 1.1) glaucoma medications which decreased postoperatively to 13.0 mm Hg (95% CI 11.5 to 14.4) and 1.0 (SD 1.1) medications at 24 months (p<0.001). Success rate was 89.2% at 24 months of follow-up, and complication rate was 2.9%.

**Conclusion:**

At 24 months of follow-up, our results for GATT in PXG demonstrate that this conjunctival sparing procedure effectively lowers IOP and reduces the medications with a low complication rate, in this relatively aggressive glaucoma subtype.

## INTRODUCTION

Pseudoexfoliation syndrome (PXS) is an age-related systemic disease with primarily ocular manifestations characterised by extracellular deposition of a fibrillar, whitish-grey proteinaceous substance on the lens, iris, ciliary epithelium, corneal endothelium, trabecular meshwork and extraocular tissues.^1^ PXS is associated with numerous extraocular conditions^2^ and is also a strong independent risk factor for glaucoma in patients with ocular hypertension.^3^

Pseudoexfoliative glaucoma (PXG) is the most common form of secondary glaucoma worldwide^2^ with a worse prognosis than primary open-angle glaucoma (POAG), as patients may experience larger intraocular pressure (IOP) fluctuations, greater visual field loss and optic nerve head damage, reduced response to medications and faster progression.^4^ Medical or laser treatment is recommended as the first-line therapy, but surgical treatment is often required.^5^

Minimally invasive glaucoma surgeries (MIGS) are emerging as an alternative to trabeculectomy,^6^ which remains the gold standard procedure for lowering IOP surgically.^7^ Trabeculotomy reduces the aqueous outflow resistance through Schlemm’s canal by making an incision in the trabecular meshwork, avoiding bleb formation. The most frequent approach is *ab externo*, requiring extensive conjunctival and scleral surgery, similar to standard trabeculectomy.^8^ An *ab interno* approach, via a clear corneal incision and aided by gonioscopy, preserves the conjunctiva and sclera and maintains the option of subsequent filtration surgery.^9^

With this prospective study, we aim to present the outcomes of an *ab interno* approach, the gonioscopy-assisted transluminal trabeculotomy (GATT), in eyes with PXG with a follow-up of up to 2 years.

## MATERIALS AND METHODS

This was a prospective, interventional, non-comparative case series of 103 eyes of 84 patients diagnosed with PXG, who showed glaucoma progression despite maximal tolerated medical therapy. All GATT procedures were performed by a single experienced surgeon (ES) during January 2016 and May 2018. The study adhered to the tenets of the Declaration of Helsinki, and Institutional Review Board/Ethics Committee approval was obtained from the Commission Cantonale d’Ethique, Canton of Vaud, Switzerland. All participants provided written informed consent.

Patient demographic data was documented preoperatively as well as medications and best-corrected visual acuity (BCVA), slit-lamp biomicroscopy, gonioscopy, funduscopy and IOP measurement by Goldmann applanation tonometry. Presence of pseudoexfoliative material in the angle, lens or at the pupillary border was confirmed during the preoperative examination. Visual field perimetry and optical coherence tomography of the optic nerve head were used to establish the diagnosis of glaucoma.

### Surgical procedure

Topical 2% pilocarpine was applied preoperatively. A standard sterile procedure with application of 5% iodine drops for 3 min was performed. A paracentesis was made in the superonasal or inferonasal quadrant and viscoelastic (HEALON GV, J&J Vision, Florida, USA) was injected into the anterior chamber, and a temporal paracentesis was created. The iridocorneal angle was visualised nasally using a Swan Jacob gonioscope. Using a crescent-shaped goniotomy blade, a horizontal incision of 1–2 mm was made into the trabecular meshwork. Either an illuminated catheter (iTRACK 250A; iScience Interventional, Menlo Park, California, USA) or a thermally blunted 5.0 polypropylene thread was introduced via a separate paracentesis into this goniotomy incision and advanced parallel to the iris plane, within Schlemm’s canal, using microsurgical forceps (diamondised, end-gripping forceps of 25 Ga, FCI Ophtalmics, Paris, France). The catheter or the thread was then externalised into the anterior chamber, thereby creating a 360° *ab interno* trabeculotomy. If a 360° goniotomy was not possible mainly due to blockage of the microcatheter, the device was inserted in the opposite direction. The viscoelastic was washed out from the anterior chamber using an irrigation-aspiration system to remove blood, and then new viscoelastic was again inserted to obtain approximately a 25% anterior chamber fill. The corneal incisions were sutured, and subconjunctival steroids were injected at the end of the procedure. If cataract surgery was needed, the GATT procedure was performed immediately after standard phacoemulsification and intraocular lens implantation.

### Postoperative protocol

A regimen of topical medications was prescribed as follows. A combination of topical dexamethasone and chloramphenicol (Spersadex comp., Théa Pharma, Clermont-Ferrand, France) for 4 weeks, starting with every three hours during the first week after surgery and then tapering to four times per day the second week and two times per day the third week and one time per day the fourth week. Pilocarpine 2% (Spersa-carpine, OmniVision AG, Neuhausen am Rheinfall, Switzerland) three times per day for 1 month and diclofenac 0.1% (Dicloabak, Théa Pharma, Clermont-Ferrand, France) three time per day for 2 months. All glaucoma medications were routinely stopped at day 1, unless there was an IOP spike or steroid response during follow-up.

Postoperative examinations were performed at day 1, week 1, week 3, week 6, month 3, month 6, month 12, month 18 and month 24, or more frequently if clinically indicated by the physician. BCVA, IOP, number of glaucoma medications and all complications were recorded.

### Outcome measures

Primary outcomes were IOP and the number of glaucoma medications. Secondary outcomes were success rate, defined as IOP reduction ≥20% from baseline and IOP between 6 and 21 mm Hg without further glaucoma surgery, and complication rate. Hyphaema was defined as any blood seen in the anterior chamber (AC) and was only considered a complication when it required washout, either because it did not resolve in the first 2 weeks or if there was a complete hyphaema. IOP spikes (IOP≥26 mm Hg) were only considered a complication when they were present on two consecutive visits and lasted at least 2 weeks, defined as transient hypertony. Transient hypotony was defined as IOP lower than 6 mm Hg on two consecutive visits more than 2 weeks apart or when low IOP was accompanied by maculopathy, a shallow or flat anterior chamber, or choroidal effusions/haemorrhage.

Statistical analysis was performed with R version 3.6.1 (R Core Team 2019. R: A language and environment for statistical computing. Foundation for Statistical Computing, Vienna, Austria). Normally distributed variables were reported as means alongside SD and 95% CIs; non-normal variables were reported as median and IQRs. Means of two continuous normally distributed variables were compared by independent samples Student’s t-test. Patients lost to follow-up were censored at their last visit. Mean IOP and number of medication reductions between preoperative value and postoperative value at a time point were calculated only with the patients who had undergone a visit at that time point.

## RESULTS

Patient demographics are shown in table 1.

**Table 1:** Demographic information

	n	Mean values
Number of eyes (patients)	103 (84)	
Age in years (range)		75.5 (8.9) (51–92)
Sex		
Women	43 (51.2%)	
Men	41 (48.8%)	
Lens status		
Phakic	50	
Pseudophakic	53	
BCVA (logMAR)		0.3 (0.3)
MD		11.9 (8.3)
Prior glaucoma procedures	11 (10.7%)	
Trabeculectomy	1	
Trabeculectomy+iStent	1	
iStent	3	
Deep sclerectomy	6	

BCVA, best-corrected visual acuity; logMAR, logarithm of the Minimum Angle of Resolution; MD, mean deviation of 24-2 visual field test.

**Figure 1:**
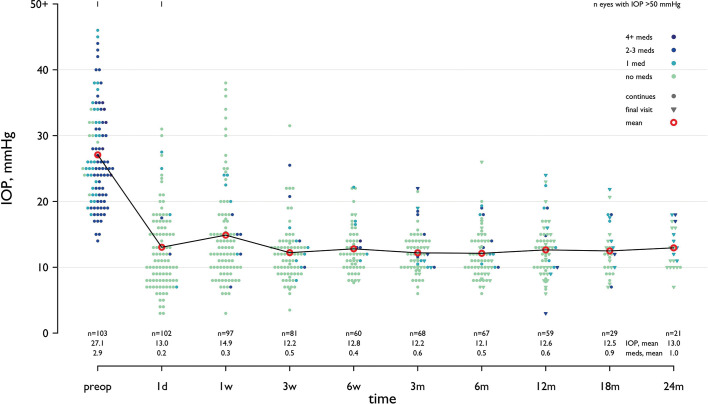
Intraocular pressure (IOP) and number of medications at each time point over 24 months of follow-up. Each eye is represented with different colours according to number of medications and with different symbols if continuing visit (dot) or if the time point represented the last visit (censoring). The number of eyes and mean values are shown on the abscissa. The red circle represents the mean IOP value at each time point. d, days; m, months; w, weeks.

**Figure 2:**
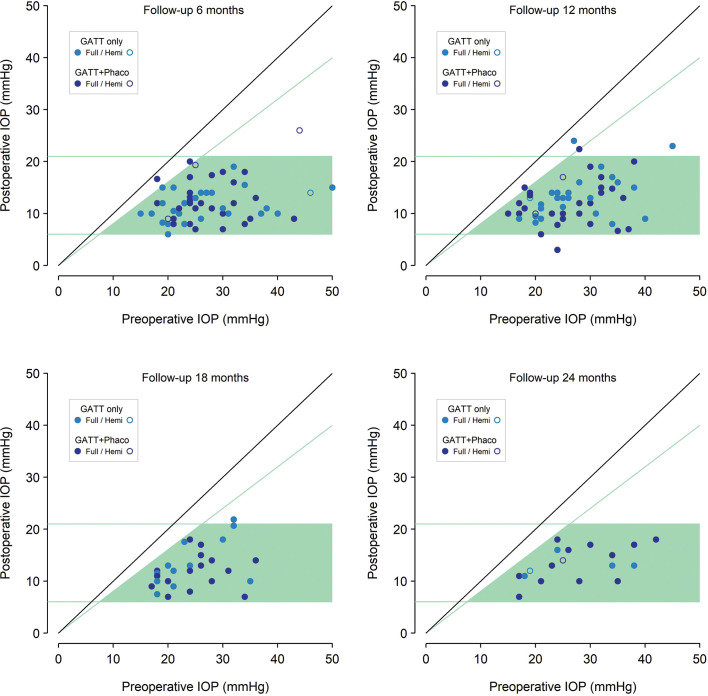
Scattergram of preoperative intraocular pressure (IOP) value (on the abscissa) and postoperative value (on the ordinate) at 6 months, 12 months, 18 months and 24 months of follow-up. The diagonal 45° line (solid black) indicates no change. Values between cut-off green lines at 6 and 21 mm Hg and under the diagonal that represents a 20% IOP decrease are classified as surgical success. Successful cases are spotted over a light green background.

Combined cataract surgery with GATT was performed in 50 eyes while the remaining 53 eyes were already pseudophakic. Postoperative IOP was significantly lower than baseline at all study time points for both groups, and there was no statistically significant difference between both groups (p=0.652, Mann-Whitney U test). There were no significant differences in glaucoma medications between groups at any study time (p=0.179, two-sample t-test). For all cases in this study, mean preoperative IOP was 27.1 (95% CI 25.5 to 28.7) mm Hg using 2.9 (SD 1.1) glaucoma medications. Postoperatively, IOP was 13.0 (95% CI 11.6 to 14.4) mm Hg at day 1, which corresponds to an IOP reduction of 52% with a mean IOP of 14.1 (95% CI 12.1 to 16.0) mm Hg. Details of preoperative and postoperative IOP and medication burden throughout follow-up are presented in table 2 and figures 1 and 2.

**Table 2:** Intraocular pressure and medications: mean values through follow-up

		Intraocular pressure mean (95% CI)			Medications mean (SD)		
Time	n	Preoperative mm Hg	Postoperative mm Hg	Reduction mm Hg (%)	Preoperative #	Postoperative #	Reduction #
All cases							
1 d	102	27.1 (25.5–28.7)	13.0 (11.6–14.4)	14.1 (12.1–16.0) (52)	2.9 (1.1)	0.2 (0.8)	2.7 (1.4)
1 w	97	27.2 (25.6–28.9)	14.9 (13.4–16.4)	12.3 (10.3–14.4) (45)	2.8 (1.1)	0.3 (0.9)	2.5 (1.5)
3 w	81	27.0 (25.1–28.9)	12.2 (11.2–13.2)	14.8 (12.7–16.9) (55)	2.8 (1.1)	0.5 (0.9)	2.4 (1.5)
6 w	60	27.2 (25.2–29.2)	12.8 (11.9–13.7)	14.4 (12.4–16.4) (53)	2.9 (1.1)	0.4 (1.0)	2.5 (1.6)
3 m	68	27.7 (25.7–29.8)	12.2 (11.4–13.0)	15.5 (13.5–17.6) (56)	2.8 (1.2)	0.6 (1.1)	2.2 (1.6)
6 m	67	27.0 (25.0–29.0)	12.1 (11.2–13.0)	14.9 (12.9–16.9) (55)	2.9 (1.1)	0.5 (1.0)	2.3 (1.4)
12 m	59	26.7 (24.9–28.5)	12.6 (11.6–13.7)	14.1 (12.3–15.8) (53)	2.9 (1.1)	0.6 (1.0)	2.3 (1.1)
18 m	29	24.6 (22.4–26.8)	12.5 (11.0–14.0)	12.1 (10.0–14.2) (49)	3.0 (0.8)	0.9 (1.2)	2.1 (1.6)
24 m	21	26.3 (22.8–29.9)	13.0 (11.5–14.4)	13.4 (10.4–16.4) (51)	2.7 (0.9)	0.9 (1.1)	1.8 (1.3)
GATT only							
1 d	53	26.8 (24.7–29.0)	12.6 (11.1–14.0)	14.3 (11.7–16.9) (53)	3.0 (1.2)	0.3 (0.9)	2.8 (1.4)
1 w	50	27.0 (24.8–29.2)	14.2 (12.1–16.3)	12.8 (10.0–15.6) (47)	2.9 (1.2)	0.2 (0.7)	2.7 (1.4)
3 w	39	25.9 (23.4–28.5)	12.7 (11.3–14.2)	13.2 (10.1–16.3) (51)	2.9 (1.2)	0.5 (1.0)	2.5 (1.6)
6 w	31	27.5 (24.5–30.4)	13.2 (11.8–14.5)	14.3 (11.1–17.5) (52)	3.2 (1.2)	0.7 (1.3)	2.5 (1.8)
3 m	33	26.9 (24.3–29.5)	13.1 (11.8–14.3)	13.9 (11.6–16.2) (52)	3.0 (1.2)	0.9 (1.4)	2.1 (1.6)
6 m	33	26.8 (24.2–29.3)	12.3 (10.8–13.7)	14.5 (12.0–17.1) (54)	3.1 (1.1)	0.7 (1.1)	2.4 (1.4)
12 m	32	27.2 (24.6–29.7)	13.3 (11.7–15.0)	13.8 (11.3–16.3) (51)	2.9 (1.1)	0.9 (1.2)	2.0 (1.0)
18 m	15	25.0 (21.7–28.3)	11.8 (9.9–13.8)	13.2 (9.8–16.5) (53)	3.0 (0.8)	1.0 (1.3)	2.0 (1.6)
24 m	8	26.5 (19.9–33.1)	13.0 (10.7–15.3)	13.5 (7.6–19.4) (51)	3.0 (0.8)	0.9 (0.9)	2.1 (1.0)
Combined GATT and cataract surgery							
1 d	49	27.4 (24.8–29.9)	13.6 (11.0–16.1)	13.8 (10.8–16.9) (50)	2.7 (1.1)	0.2 (0.7)	2.6 (1.3)
1 w	47	27.5 (24.9–30.1)	15.6 (13.5–17.8)	11.8 (8.7–15.0) (43)	2.7 (1.1)	0.5 (1.0)	2.2 (1.5)
3 w	42	28.0 (25.1–30.9)	11.8 (10.4–13.1)	16.2 (13.3–19.2) (58)	2.7 (1.1)	0.4 (0.9)	2.3 (1.5)
6 w	29	26.9 (24.2–29.7)	12.4 (11.2–13.7)	14.5 (11.8–17.2) (54)	2.6 (0.9)	0.2 (0.6)	2.4 (1.3)
3 m	35	28.5 (25.2–31.8)	11.4 (10.5–12.3)	17.1 (13.8–20.4) (60)	2.6 (1.2)	0.3 (0.7)	2.3 (1.5)
6 m	34	27.2 (23.9–30.5)	12.0 (10.9–13.1)	15.2 (12.0–18.4) (56)	2.6 (1.1)	0.3 (0.7)	2.3 (1.4)
12 m	27	26.2 (23.6–28.9)	11.8 (10.4–13.2)	14.4 (11.8–17.0) (55)	2.8 (1.0)	0.2 (0.6)	2.6 (1.2)
18 m	14	24.1 (20.9–27.4)	13.2 (10.6–15.8)	11.0 (8.2–13.8) (46)	3.0 (0.9)	0.8 (1.1)	2.2 (1.6)
24 m	13	26.2 (21.4–31.1)	12.9 (10.8–15.0)	13.3 (9.3–17.3) (51)	2.5 (1.0)	0.9 (1.2)	1.7 (1.4)

d, days; GATT, gonioscopy-assisted transluminal trabeculotomy; m, months, n, number of cases; w, weeks; #, number of medications.

Complete GATT procedure (full 360º) was achieved in 97 of 103 eyes. In the six cases without 360º treatment, the mean IOP difference between baseline and last postoperative time point was 12.07 (CI 95% 5.64–18.49, p=0.002) and mean medication difference was −1.25 (CI 95% 0.16–2.35, p=0.029).

Mean BCVA (logMAR) was 0.3 (SD 0.3) before surgery, 0.2 (SD 0.4) at 12 months (p=0.155) and 0.4 (SD 0.8) at 24 months (p=0.608). Overall success rate was 89.2% at 24 months. Transient hyphaema was observed in all cases in the first postoperative week and was usually suspended in viscoelastic in the AC. By week 1, these had largely resolved. If hyphaema persisted at week 1, eyes were reviewed at week 2. Micro-hyphaema of <1 mm with IOP in the low teens rarely persisted beyond 2 weeks and had no long-term consequences. Conversely, if at week 1 there was a solid coagulated hyphaema filling >50% of the AC (with or without elevated IOP), we reviewed the patient at week 2 with a view to AC washout. In table 3, we reported one case such a hyphaema which required AC washout in the operating room.

**Table 3:** Complications

	n=103
Intraoperative complications	0
Hyphaema (persisting>2 weeks)	1
Transient hypotony	1
Transient hypertony	1
Retinal/choroidal complication	0

There were 25 cases with isolated IOP spikes, but only 1 case with transient hypertony as defined above. Overall complication rate was 2.9% (table 3).

## DISCUSSION

The goal of glaucoma management is to preserve the patients’ quality of life by maintaining visual field and acuity with the least possible adverse effects of therapy.^10^ It was not until the advent of MIGS procedures,^11^ which aim to lower IOP with low complication rates and minimal tissue destruction that trabeculotomy (originally described in 1960)^12^ experienced a renaissance. MIGS aim at limiting conjunctival damage and the development of bleb-less surgeries.^13^ Recent studies have underlined the importance of the cost-effectiveness of MIGS and improving patients’ quality of life.^14^

Grover *et al* reported their results for the GATT procedure in POAG,^15 16^ in primary congenital glaucoma and juvenile open-angle glaucoma^17^ and in patients with previous glaucoma surgery,^18^ and other research groups reported similar results for GATT in POAG^19–^ ^21^ and steroid-induced glaucoma.^22^ To the best of our knowledge, there are no previous studies investigating the outcomes of GATT in patients with PXG. Our results show that GATT may be even more effective in PXG than in POAG, as previously reported with limited *ab interno* trabeculectomy^23^ and in some *ab externo* trabeculotomy studies.^24 25^

In our study, the marked reduction of IOP and number of medications was statistically significant at all postoperative time points. There were no major complications^26^ in any patient, and the combination of cataract surgery and GATT maintained the same effectiveness as GATT alone in pseudophakic patients. In 6 out of 103 eyes, a complete 360-degree trabeculotomy was not achieved; however, all these partial cases were successful. Hyphaema was observed in all cases at day 1, and if persistent, this may lead to downstream complications^27^; however, it usually resolved within the first 2 weeks. Conversely, hyphaema has also been proposed as a positive prognostic factor following canaloplasty.^28^

This study has some limitations: the non-comparative design, the mid-term follow-up of up to 24 months and the thinning of our data as some patients had their procedures performed less than 2 years before the end of this study. The procedure is cost-effective, especially when using a polypropylene thread to circumnavigate Schlemm’s canal, and the cost is considerably higher when the microcatheter is used. We switched from catheter-GATT to suture-GATT due to economic reasons, after 1 year into the study; therefore, a formal comparison between the two groups was not possible due to limited follow-up. The procedure has a learning curve that requires in-depth knowledge of angle anatomy. This technique also requires an intact collector system, and currently, there is no reliable preoperative method to assess the episcleral venous flow; however, some intraoperative approaches have been suggested.^29 30^

In conclusion, our study demonstrates that GATT safely and effectively lowers IOP in PXG, either when performed alone or when performed in combination with cataract surgery. Given the marked IOP lowering effect and low complication rates, and the relatively aggressive nature of PXG, we consider GATT to be a suitable first-line treatment for PXG patients. GATT, although technically demanding, also provides a very cost-effective MIGS procedure when a polypropylene suture is used.
